# Effect of the COVID-19 Pandemic on the Psychological Health of Patients Who Underwent Liver Transplantation Due to Hepatocellular Carcinoma

**DOI:** 10.3390/diagnostics13081410

**Published:** 2023-04-13

**Authors:** Sami Akbulut, Zeynep Kucukakcali, Hasan Saritas, Cigdem Bozkir, Murat Tamer, Musap Akyuz, Nazlican Bagci, Selver Unsal, Mehmet Serdar Akbulut, Tevfik Tolga Sahin, Cemil Colak, Sezai Yilmaz

**Affiliations:** 1 Department of Surgery and Liver Transplant Institute, Inonu University Faculty of Medicine, 44280 Malatya, Turkey; 2 Department of Public Health, Inonu University Faculty of Medicine, 44280 Malatya, Turkey; 3 Department of Biostatistics, Bioinformatics and Medical Informatics, Inonu University Faculty of Medicine, 44280 Malatya, Turkey; 4 Department of Surgical Nursing, Siirt University Faculty of Health Science, 56100 Siirt, Turkey; 5 Department of Nutrition and Dietetics, Inonu University Faculty of Health Science, 44280 Malatya, Turkey; 6 Department of Surgical Nursing, Inonu University Faculty of Nursing, 44280 Malatya, Turkey; 7 Department of Nursing Service, Inonu University Faculty of Medicine, 44280 Malatya, Turkey; 8 Department of Social Work, Bingol State Hospital, 12000 Bingol, Turkey

**Keywords:** liver transplantation, hepatocellular carcinoma, COVID-19 pandemic, Depression Anxiety Stress Scale, Coronavirus Anxiety Scale, COVID-19 vaccine, vaccine awareness

## Abstract

Background: The primary aim of this study was to compare liver transplant (LT) recipients with and without hepatocellular carcinoma (HCC) in terms of COVID-19-related depression, anxiety, and stress. Method: A total of 504 LT recipients with (HCC group; n = 252) and without HCC (non-HCC group; n = 252) were included in the present case–control study. Depression Anxiety Stress Scales (DASS-21) and Coronavirus Anxiety Scale (CAS) were used to evaluate the depression, stress, and anxiety levels of LT patients. DASS-21 total and CAS-SF scores were determined as the primary outcomes of the study. Poisson regression and negative binomial regression models were used to predict the DASS and CAS scores. The incidence rate ratio (IRR) was used as a coefficient. Both groups were also compared in terms of awareness of the COVID-19 vaccine. Results: Poisson regression and negative binomial regression analyses for DASS-21 total and CAS-SF scales showed that the negative binomial regression method was the appropriate model for both scales. According to this model, it was determined that the following independent variables increased the DASS-21 total score: non-HCC (IRR: 1.26; *p* = 0.031), female gender (IRR: 1.29; *p* = 0.036), presence of chronic disease (IRR: 1.65; *p* < 0.001), exposure to COVID-19 (IRR: 1.63; *p* < 0.001), and nonvaccination (IRR: 1.50; *p* = 0.002). On the other hand, it was determined that the following independent variables increased the CAS score: female gender (IRR:1.75; *p* = 0.014) and exposure to COVID-19 (IRR: 1.51; *p* = 0.048). Significant differences were found between the HCC and non-HCC groups in terms of median DASS-21 total (*p <* 0.001) and CAS-SF (*p* = 0.002) scores. Cronbach’s alpha internal consistency coefficients of DASS-21 total and CAS-SF scales were calculated to be 0.823 and 0.783, respectively. Conclusion: This study showed that the variables including patients without HCC, female gender, having a chronic disease, being exposed to COVID-19, and not being vaccinated against COVID-19 increased anxiety, depression, and stress. High internal consistency coefficients obtained from both scales indicate that these results are reliable.

## 1. Introduction

The most common primary liver cancer is hepatocellular carcinoma, and it ranks as the fifth most common cause of cancer-related deaths [1,2]. The incidence of hepatocellular carcinoma (HCC) is highest in developing countries. The risk factors include hepatitis B virus (HBV), hepatitis C virus (HCV), nonalcoholic fatty liver disease (NAFLD), alcohol-induced cirrhosis, smoking, obesity, diabetes, iron overload, and exposure to various dietary factors [1,3]. Patients with chronic liver disease have an especially high risk of developing HCC. Therefore, a routine surveillance program is necessary [4]. HCC is diagnosed incidentally, and it is usually in advanced stages in patients with chronic liver disease (CLD) who are not a part of a routine follow-up program. For this reason, the prognosis of HCC is generally poor [4].

HCC is a serious public health problem especially in underdeveloped and developing countries [4]. The incidence is rising, and HCC has become one of the leading causes of cancer-related deaths worldwide [5,6]. Current research has enabled us to develop strategies for prevention and early diagnosis while helping immensely in elucidating the risk factors, epidemiology, and molecular and genetic profiles of HCC [7,8]. There are many treatment modalities used for different indications in HCC, which include surgical resection, radio-frequency ablation (RFA), microwave ablation (MWA), cryoablation, high-intensity focused ultrasound (HIFU) ablation, irreversible electroporation (IRE), transcatheter arterial chemoembolization (TACE), transcatheter arterial radioembolization (TARE), external radiotherapy, systemic therapy (immunotherapy, antiviral therapy, and molecular targeted therapy), and liver transplantation (LT), which are used in combination in the treatment of HCC [9,10]. The success of these treatment options depends on the underlying primary liver disease and the characteristics of the tumor. A significant portion of HCC patients present in moderate to advanced stages in which a definitive therapy is not possible. This emphasizes the importance of early diagnosis for the effective treatment of HCC [5,11].

LT has become the gold standard for the definitive treatment of many liver diseases, especially end-stage CLD, primary liver tumors, and acute liver failure [12,13]. HCC mostly develops more frequently in a cirrhotic liver, and LT performed in such cases offers patients a long-term survival, as it removes both the tumor and the cirrhotic microenvironment [14,15,16]. Therefore, LT is the best treatment option for HCC, and HCC cases account for 15–50% of all liver transplants performed in most of the transplant centers [16].

Patients with LT for any reason need special care, starting with the preoperative preparation phase and ending with postoperative care after the surgical intervention. It is very important to prevent organ rejection by administering immunosuppressive medications in patients undergoing LT. Immunosuppression created in LT recipients makes patients vulnerable to many opportunistic and other infections [17].

The severe acute respiratory syndrome coronavirus 2 (SARS-CoV-2) pandemic that emerged in China toward the end of 2019 soon became a global concern, infecting 752,517,552 people worldwide by January 2023. The infectious disease caused by SARS-CoV-2 was referred to as coronavirus infectious disease 2019 (COVID-19). Many studies have shown that, in addition to having a higher risk of contracting severe COVID-19, the patients with chronic health conditions had a higher risk of mortality due to COVID-19 [18]. Compared to the healthy population, patients with CLD, hepatobiliary malignancies, and LT recipients are more likely to contract the SARS-CoV-2 virus, as the innate immune system is severely suppressed. Furthermore, these patients also have higher risk mortality due to COVID-19. Therefore, it was vital for patients who received LT to strictly comply with infection control measures during the pandemic [17].

The restrictions that came into our lives during the COVID-19 pandemic paved the way for the development of mental disorders such as anxiety, stress, depression, and posttraumatic stress in individuals all around the world [19,20]. Both the immunosuppressive and psychosocial effects of the drugs used and the difficulty in adapting to the restrictive lifestyle resulted in pandemic-related anxiety, stress. and depression symptoms of LT recipients with severity varying from patient to patient during the pandemic period. In addition, potentially life-threatening complications such as graft rejection and cardiovascular diseases may also occur after LT. The quality of life of patients before LT is impaired, and their quality of life may be affected due to the expected posttransplant complications. This situation poses a high risk for somatization and mood disorders in patients [21,22]. In addition to all these postoperative complications, the fear of contracting SARS-CoV-2 may cause additional mood disorders and may reveal high stress and anxiety elements [23].

Very important infectious diseases have threatened humans in the past. In the past, polio, measles, smallpox and many more infections have been managed effectively by the development of effective vaccines [24]. However, vaccine hesitancy has consistently been a major problem in preventive medicine. Vaccine hesitancy is defined as denial of the vaccine as a result of distrust of the vaccine or the manufacturer. Moreover, those individuals who are hesitant to take the vaccine may have a different perception of the seriousness of the disease itself [25]. Confidence in the vaccine and vaccine hesitancy is an important health problem globally, and it has long been so even before the emergence of SARS-CoV-2. Hence, in 2019, World Health Organization (WHO) declared vaccine hesitancy to be a major health problem of the era [26]. Together with the addition of COVID-19, this contradiction resulted in further controversies for the developed vaccines in an emergency setting. The problems mainly stem from the vast amount of complex knowledge regarding the efficacy and side effects of the vaccines [26]. In our opinion, one of the major concerns regarding the vaccines is understanding the mechanism of action that is provided. Currently, it seems that vaccines do not prevent the transmission of the disease but reduce the risk of contracting the severe form of COVID-19.

The vaccine acceptance rates show geographic, ethnic, and religious differences [25]. Generally, the rate varies between nearly 70 and 90% in studies from different eras [26,27]. A population-based controlled study showed that the vaccine rejection rate in Turkey was 3% of the population, and the hesitancy rate was 34% in the 3936 participants. This decision was mainly governed by the disbelief toward the natural origins of the virus [28]. Another study of 384 participants (selected from a well-educated population) enrolled in Istanbul showed a vaccine hesitancy rate of 45% [29]. Furthermore, Ikiisik et al. [30] have analyzed the vaccine hesitancy among the healthcare workers in the Istanbul district and have shown that 29% were unwilling and another 21% refused to be vaccinated. In our opinion, LT recipients and cancer patients form a special subgroup of the general population. Patients with HCC who have received a LT fit both criteria (i.e., being a solid organ transplant recipient and also being a cancer patient). These patients have been gifted a second chance and therefore may take health issues more seriously. Investigation of vaccine hesitancy and vaccination rates among this population would yield very beneficial information. For this reason, one of the aims of our study was to evaluate the vaccination and vaccine hesitancy rates in this patient group.

**H_0_:**  *There is no significant difference between LT patients with and without HCC regarding COVID-19-related mental health conditions.*

**H_1_:**  *There is a difference between groups regarding COVID-19-related mental health conditions, and the difference is more pronounced in patients with HCC.*

**H_2_:**  *There is a difference between groups regarding COVID-19-related mental health conditions, and the difference is more pronounced in patients without HCC.*

## 2. Materials and Methods

### 2.1. Type, Place, and Duration of Research

Patients who underwent LT for any reason at Inonu University Liver Transplantation Institute in Malatya, Turkey, between March 2002 and July 2021 and who survived until the end of the study duration (October 2021) were determined as the main population for this survey-based descriptive and cross-sectional study. Before the study began, study approval was obtained from the Directorate of Liver Transplantation Institute (2021/93879).

### 2.2. Study Protocol and Ethics Committee Approval

This study involving human participants was conducted in accordance with the ethical standards of the institutional and national research committee and with the 1964 Helsinki Declaration and its later amendments or comparable ethical standards. Ethical approval was obtained from the Inonu University Institutional Review Board (IRB) for Non-Interventional Clinical Research (Approval No: 2021/2553). The STROBE (Strengthening the Reporting of Observational Studies in Epidemiology) guideline was used for the current study [31].

### 2.3. The Population of the Research and Determination of the Groups

Based on the electronic patient information and management system data used in our transplant institute, 273 patients with HCC who underwent LT due to HCC in our institute until July 2021 and were alive as of October 2021 were enrolled as the study group (HCC group). In the subsequent detailed analysis, 21 patients with HCC were excluded from the study for the following reasons: died in other centers despite appearing to be alive in our system (n = 16), were <18 years old (n = 4), and could not be reached by phone (n = 1). The remaining 252 HCC patients were assigned to the HCC group. It was determined that 1571 patients were ≥18 years and underwent LT for non-HCC etiologies during the same period. With the simple randomization function [i.e., RAND()] in Microsoft Excel, 252 patients who randomly matched the HCC group were selected, and these patients were assigned to the control group (non-HCC group). Two researcher groups were formed for the telephone interviews, and each group included three authors experienced in survey studies. The questionnaire form was filled using the telephone interview technique. Detailed information regarding the questionnaire form is given below.

### 2.4. Inclusion and Exclusion Criteria

This study was a questionnaire-based study, and, therefore, patients aged ≥18 years old who had sufficient Turkish language to read and understand the questionnaire questions were chosen. Patients transplanted for HCC were included in the study group and defined as the HCC group, and patients who received LT for non-HCC reasons were included in the control group and defined as the non-HCC group. Patients who were already discharged from the hospital after LT and have a postoperative follow-up of at least 3 months were found to be eligible for the study. Patients who did not want to participate in the study or did not mark a part of the questions were excluded from the study and the evaluation. Fortunately, we had only one patient who did not complete the questionnaire forms excluded from the study.

### 2.5. Parameters and Scales Used in the Study

#### 2.5.1. Sociodemographic Characteristics Form

The questionnaire used in this study consists of 25 questions and 2 scales. The questions related to the clinical and sociodemographic characteristics of the study were briefly defined as follows: age, gender, height, weight, marital status, educational level, residency, monthly income, smoking, presence of chronic disease (diabetes mellitus, hypertension, asthma, chronic obstructive pulmonary disease, cardiopulmonary disease), immunosuppressive medications (tacrolimus, everolimus, steroid, mycophenolate mofetil), exposure to COVID-19, initiation of antiviral treatment for COVID-19, hospitalization due to COVID-19 (service, intensive care unit), vaccination status (Sinovac™, BionTech™, both, none), vaccine dose (one, two, three), postvaccination COVID-19 exposure, COVID-19 vaccine hesitancy, belief in the protective effects of the COVID-19 vaccine, opinion on legally mandating the vaccine, level of knowledge of healthcare professionals about COVID-19, and requirement for any tapering in the dose and type of immunosuppressive medications when COVID-19 was contracted.

#### 2.5.2. Coronavirus Anxiety Scale-Short Form (CAS-SF)

CAS-SF, which aims to determine the severity of anxiety caused by the COVID-19 pandemic in society, was first defined by Lee in 2020 [32]. According to the study by Lee and colleagues, the factor loads of the items of the CAS-SF scale ranged from 0.81 to 0.88, while the Cronbach’s alpha coefficient for the scale was calculated as 0.93. The validity and reliability tests of the Turkish version of this scale were performed by Bicer and colleagues in 2020 [33]. Bicer and colleagues [33] demonstrated that the factor loads of the items in the Turkish version of the CAS-SF scale, which consisted of one dimension and 5 items, varied between 0.63 and 0.78. Bicer and colleagues calculated the Cronbach’s alpha coefficient of this scale to be 0.83. The responses in the CAS-SF scale include included five-point Likert type questions which are as follows: not at all (0 points), rare, less than one or two days (1 point), several days (2 points), more than seven days (3 points), and nearly every day over the last two weeks (4 points). The minimum points that can obtained from this scale is 0 while the maximum is 20. Lee and colleagues calculated an optimal cutoff point for anxiety (≥9 points) using receiver operating curve (ROC) analysis and calculated the sensitivity and specificity values of this cutoff point to be 90% and 85% (area under the curve [AUC]: 0.94; *p* < 0.001), respectively. A score of 9 and above supported the presence of anxiety related to COVID-19.

#### 2.5.3. Depression, Stress and Anxiety Scale-Short Form (DASS-21)

The long form of the DASS scale, developed by Lovibond and Lovibond [34], includes 42 items and three subdimensions. The Cronbach’s alpha internal consistency coefficients for the depression, anxiety, and stress subdimensions of the long version of the DASS scale were calculated as 0.91, 0.84, and 0.90, respectively. Henry and Crawford [35] designed the DASS-21 scale, which is a short version of the DASS scale. They hypothesized that the short form is also valid for the same measurement. The Cronbach’s alpha reliability coefficient of the DASS-21 form was calculated as 0.93. In terms of the subdimension analysis, Cronbach’s alpha coefficients of the depression, anxiety, and stress subdimensions for the DASS-21 were calculated to be 0.88, 0.82, and 0.90, respectively. The Turkish adaptation of the DASS-21 form was made by Yılmaz and colleagues [36] in 2017. The DASS-21 scale consisted of four-Likert type questions. The choices were as follows: never (0 points; did not apply to me at all), sometimes (1 point; applied to me to some degree or some of the time), often (2 point; applied to me to a considerable degree or a good part of the time), and almost always (3 point; applied to me very much or most of the time). There were seven questions in each of the depression (3, 5, 10, 13, 16, 17, 21), anxiety (2, 4, 7, 9, 15, 19, 20) and stress (1, 6, 8, 11, 12, 14, 18) subdimensions. The Cronbach’s alpha reliability and internal consistency coefficient of the DASS-21 scale was 0.87 [36]. In terms of subdimension analysis, Cronbach’s alpha internal consistency coefficients of depression, anxiety, and stress were calculated to be 0.82, 0.81, and 0.76, respectively. There are no reverse scored items in the scale.

### 2.6. Statistical Analysis

IBM SPSS Statistics 25.0 (Statistical Package for the Social Sciences, Inc, Chicago, IL, USA) program was used in the analysis. The Kolmogorov–Smirnov test of normality was used to determine whether the variables had a normal distribution. Quantitative data were given as a median and interquartile range (IQR). Qualitative variables were given as the number of affected individuals and the percentage of the study population. For statistical analyses, the Mann–Whitney U test, Kruskal–Wallis test, Pearson chi-square test, and Yates corrected chi-square test and Spearman rho correlation coefficient were used. The Conover test was used for pairwise comparisons of the quantitative variables. The effect sizes for each test were calculated. The effect size (Cohen d) for the Mann–Whitney U test is interpreted as a small effect between 0.20 and 0.50, a moderate effect between 0.50 and 0.80, and a large effect above 0.80. For the Kruskal–Wallis test, the effect size (Cohen d) is interpreted as a small effect between 0.10 and 0.25, a medium effect between 0.25 and 0.40, and a large effect with values above 0.40. For chi-square tests, the effect size is interpreted as a small effect between 0.10 and 0.30, a medium effect between 0.30 and 0.50, and a large effect above 0.50 [37]. In cases where the dependent variable is continuous, linear regression analysis is performed using the least squares method to examine the relationship between the variables. However, if the dependent variable is discrete or count data (countable quantities or counting variable), the analyses using linear regression models would yield ineffective, inconsistent, and contradictory results. Therefore, different regression models have been developed for count data. Among them, the most well-known regression models are the Poisson and negative binomial regression models. The values of the survey results applied in the study are count variables (individual pieces of count data), and the relationship between the DASS-21 total and CAS-SF scores and other variables was examined with Poisson and negative binomial regression methods. In the regression models, the output variables were DASS-21 total and CAS-SF scores, and the input variables were the groups (HCC, non-HCC), age, gender, educational status, income status, smoking, presence of chronic disease, the status of COVID-19 exposure, and the status of COVID-19 vaccination. The Akaike information criterion (AIC) was used for the comparison of the different widely used models. The smallest calculated AIC value was used in this model. Regression analyses were performed with the R program. For Poisson regression analysis, the glm function was used, and for negative binomial regression analysis, the glm.nb function included in the MASS package [38] was used. Goodness-of-fit test for negative binomial regression was performed with the “epiDisplay” of the R package. This function is used to test whether any data conforms to the negative binomial distribution. In order to compare the regression models, AIC values were calculated using the stargazer function included in the stargazer package [39]. A *p* < 0.05 was considered to be a statistically significant level.

A two-way ANOVA analysis was performed to determine whether sociodemographic characteristics (gender, chronic disease, marital status, monthly income, educational levels, residency) had an effect on the relationship between DASS-21 total and CAS-SF variables and the group variable (HCC vs. non-HCC).

In the multilayer perceptron method (MLP), which is a type of feed-forward artificial neural network from machine learning methods, group (HCC and non-HCC), gender, chronic diseases, exposure to COVID-19, vaccination for COVID-19, hesitancy to vaccinate for COVID-19, and belief in the efficacy of the COVID-19 vaccine variables were taken as input variables, and DASS-21 total and CAS-SF were taken as output variables. Activation functions were hyperbolic tangent in the hidden layer(s) and identity in the output layer. The number of hidden layers and number of units in the hidden layer was 1, and the number of units and rescaling method for scale dependents was 1 and standardized, respectively. During the modeling phase, 70% of the data set was used for training and 30% for test data. The sum of squares error and relative error were estimated to evaluate the model performance.

## 3. Results

This study included 504 patients ranging in age from 20 to 80 years (median: 57 years). In all, 369 (73.2%) of the patients were male, whereas 135 (26.8%) were female. The age of the patients in the HCC group ranged from 20 to 80 years (median: 60 years), whereas the age of the patients in non-HCC group ranged from 20 to 73 years (median: 55 years). While 213 (84.5%) of HCC patients were males and 39 (15.5%) were females, 156 (61.9%) of the non-HCC patients were males and 96 (38.1%) were females.

According to the results of the two-way ANOVA analysis, the gender (*p* = 0.655), educational level (*p* = 0.962), monthly income (*p* = 0.773), residency (*p* = 0.139), chronic disease (*p* = 0.972), and marital status (*p* = 0.391) variables did not contribute to any difference in the relationship between DASS-21 total and the group variable. Moreover, the gender (*p* = 0.382), educational level (*p* = 0.495), monthly income (*p* = 0.060), residency (*p* = 0.176), chronic disease (*p* = 0.632), and marital status (*p* = 0.731) variables did not contribute to any difference in the relationship between CAS-SF and the group variable.

The comparison of the HCC and non-HCC groups in terms of sociodemographic features are given in Table 1. In brief, statistically significant differences were found between the groups in terms of gender (*p* < 0.001), marital status (*p* = 0.009), educational level (*p* = 0.018), monthly income (*p* < 0.001), place of residence (*p* < 0.001), and presence of chronic disease (*p* = 0.008). The effect size of the significant variables suggests that they are in the small effect size category.

The analyses of the parameters related to COVID-19 and vaccines/vaccination in the HCC and non-HCC groups are given in Table 2. There were statistically significant differences between the groups in terms of COVID-19 vaccination status (*p* = 0.026), number of vaccine doses (*p* = 0.001) administered, vaccine type (*p* = 0.019), belief in the protection of the COVID-19 vaccine (*p* = 0.016), and opinion about making the COVID-19 vaccine legally mandatory (*p* < 0.001). On the other hand, there was no statistically significant relationship between the groups in terms of exposure to COVID-19, need for hospitalization due to COVID-19, postvaccine COVID-19 exposure status, or hesitancy regarding the COVID-19 vaccine.

The results regarding the effects of independent categorical variables on DASS-21′ total, stress, depression, and anxiety scores are given in Table 3. According to Table 3, a statistically significant difference was found in terms of the DASS-21 stress (*p* = 0.002), DASS-21 depression (*p* < 0.001), DASS-21 anxiety (*p* < 0.001), and DASS-21 total (*p* < 0.001) scores between the HCC and non-HCC groups. The effect size for DASS-21 anxiety suggests that it has a medium effect. In the rest of the scales, the effect sizes suggested a small effect. Analyses of variables including gender, presence of chronic disease, contracting COVID-19, being vaccinated against COVID-19, type of vaccine, presence of hesitation about the COVID-19 vaccine, and belief in the efficacy of the COVID-19 vaccine showed that a statistically significant difference was found for DASS-21 stress, DASS-21 depression, DASS-21 anxiety, and DASS-21 total scores. The effect sizes for these variables suggested a moderate effect except for the variable of hesitation to vaccinate for COVID-19. However, the effect size for hesitation to vaccinate for COVID-19 suggested a strong clinical effect. The results of the pairwise comparison analyses for the vaccine type with regard to DASS-21 stress scores suggested that there were significant differences in scores among the individuals who chose Sinovac™ versus those who chose both and those individuals who chose BioNTech™ versus those who chose both. In the DASS-21 anxiety scale, there were differences in scores between the categories choosing Sinovac™ and those who chose both. Likewise, for the DASS-21 depression and DASS-21 total variables, there were differences in scores between the individuals who chose Sinovac™ and those who chose both and between those individuals who chose BioNTech™ and those who chose both. In addition, according to the results of the pairwise comparison analysis for the belief in efficacy of the COVID-19 vaccine, there were difference in scores between those who said yes and no and those who said yes or no idea for the DASS-21 stress and anxiety scales. Likewise, each response category showed differences for the DASS-21 depression and DASS-21 total.

The results of the analyses comparing the results of CAS-SF scores in terms of independent variables are given in Table 4. The analyses of the CAS-SF scale scores showed statistically significant differences in terms of group, gender, presence of chronic disease, postvaccine COVID-19 status, presence of hesitation about the COVID-19 vaccine, and belief in the protectiveness of the COVID-19 vaccine. However, no significant difference was found in terms of variables related to exposure to COVID-19 disease, hospitalization due to COVID-19, being vaccinated for COVID-19, vaccine doses, or approval of mandating the COVID-19 vaccine legally. The effect sizes of the statistically significant variables were small.

Spearman’s rho correlation coefficient was used to test the relationship between the scale scores, and the results of the analyses are summarized in Table 5. There was a significant positive correlation between CAS, DASS-21, and its subdimensions (*p* < 0.05). In addition, the results of the reliability analysis of the scale scores are given with the Cronbach alpha reliability coefficient. The reliability coefficients of the DASS-21 stress, DASS-21 anxiety, DASS-21 depression, and CAS scales were calculated as 0.77, 0.78, 0.86 and 0.78, respectively. It can be seen that the reliability levels of the DASS-21 stress, DASS-21 anxiety, and CAS scales are quite high. Furthermore, the highest reliability was observed in the DASS-21 depression scale.

The response variables (DASS-based and CAS-based mental health data) had a negative binomial distribution with respect to the goodness-of-fit test. The AIC values were calculated based on the results of the Poisson regression model and negative binomial model used to estimate the total scores of DASS-21. The AIC value of the negative binomial regression model was 2752.7, and it was smaller than the AIC value of the Poisson regression model, which was 4070.6. Since the AIC value was smaller for the negative binomial regression method, it was chosen as the appropriate model. The coefficients and *p* values of the variables according to the results of the negative binomial regression model are summarized in Table 6. According to the coefficient significance obtained as a result of the negative binomial regression in Table 6, the group variable of age, gender, presence of chronic disease, exposure to COVID-19, and the status of vaccination for COVID-19 were found to be significant. According to the incidence rate ratio (IRR) values, being in the non-HCC group increases DASS-21 scores 1.26 times, being female 1.29 times, having a chronic disease 1.65 times, exposure to COVID-19 1.63 times, and not being vaccinated 1.50 times. In addition, a 1 unit decrease in age increases the DASS-21 scores 1.01 times.

The AIC values were calculated using the results of the Poisson regression model, and negative binomial model for estimation of the CAS-SF total scores was examined. The AIC value of the negative binomial regression model was 1054.25, and it was smaller than the AIC value of 1276.87 of the Poisson regression models. Since the AIC value was smaller for the negative binomial regression method, it was chosen as the appropriate model. The coefficients and *p* values of the variables according to the results of the negative binomial regression model are given in Table 7. Gender and exposure to COVID-19 were found to be significant as a result of our analyses. According to the incidence rate ratio (IRR) values obtained, being a woman and being infected with COVID-19 increases the CAS-SF score 1.75 times and 1.51 times, respectively.

**Figure 1:**
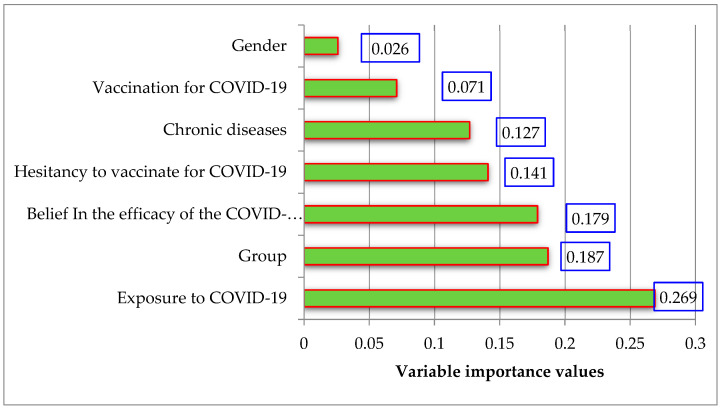
The graph of the variable importance values for DASS-21 total score.

**Figure 2:**
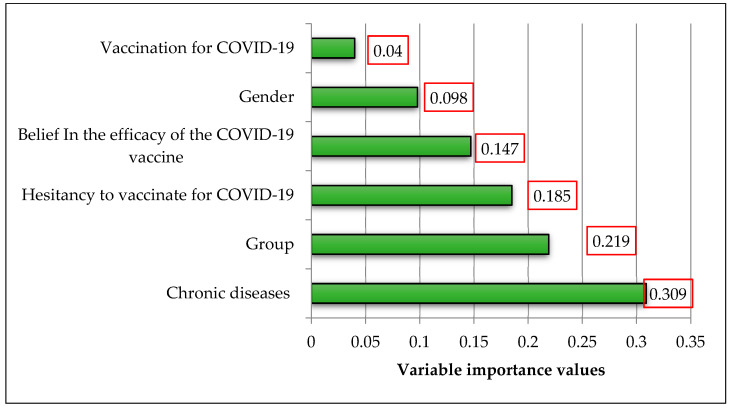
The graph of variable importance values for CAS-SF score.

According to the results from MLP model, the importance values of the input variables affecting the DASS-21 total score were determined and are presented in Table 8 and Figure 1. Among the important values, exposure to COVID-19 was the most prominent factor. As a result of the modeling, the sum of squares error was 61.08, and the relative error was 0.83. According to the results from MLP model, the importance values of the input variables affecting the CAS-SF score are given in Table 9 and Figure 2. Among the important values, chronic diseases was the most prominent factor. As a result of the modeling, the sum of squares error was 56.62, and the relative error was 0.87.

## 4. Discussion

In this study, we evaluated the factors that have an effect on depression, stress, and anxiety in individuals who received LT for HCC versus those without HCC. We used the DASS-21 and CAS-SF scales to evaluate depression, anxiety, and stress-related moods in the LT recipients. Furthermore, we also evaluated the vaccine hesitancy rate among the population. This study is unique because it was conducted on a specific and vulnerable population, and the number of individuals that were included in the study is high. In addition, evaluating the impact of COVID-19 on stress and mood-related disorders in a specific group of patients is another unique characteristic of the present study.

It is well-known that LT is a more complex procedure when compared to organ transplants in terms of both surgical technique and postsurgical follow-up. In particular, patients who undergo a living donor liver transplantation are concerned about both themselves and their donors. Therefore, the psychosocial pressure that these patients are exposed to is much higher than that of cadaveric organ transplant recipients. It sometimes takes a long time for recipients to adapt to immunosuppressive drugs and the restrictive lifestyle changes following LT. Potentially life-threatening complications such as biliary tract complications, allograft rejection, cancer recurrence, and de novo cancer development may be seen in the postoperative course of these patients [40]. For these reasons, reduced postoperative quality of life and psychosocial disorders may be higher, especially in patients. Furthermore, the presence of HCC as an indication of LT has an additional impact on the patients’ psychosocial well-being and quality of life [41]. In addition, patients with impaired quality of life in the pre-LT period (such as advanced end-stage liver disease and complications of cirrhosis that deteriorate the physiologic condition of the patient) have also deteriorated the health-related quality of life in the post-LT period. As a result, LT candidates and recipients are at high risk for anxiety, somatization, and mood disorders [21]. In our opinion, these data suggest that this subgroup of the general public is a vulnerable group that requires specific attention. Furthermore, the psychological characteristics of these patients show differences from healthy individuals.

It is crucial to prevent organ rejection in patients undergoing LT, and therefore patients have to use one or more immunosuppressive drugs throughout their lives. Such a state of immunosuppression may predispose the individuals to different types of infections, the most prominent of which are the viral and other opportunistic infections. COVID-19 has become a global crisis affecting all aspects of the society including economic and health care resources. For this reason, LT recipients are also vulnerable to COVID-19 [17]. These patients are already struggling with a disease that has psychological implications and an additional of risk of contracting a virus that has caused a global crisis that may lead to additional mood disorders. Anxiety, fear, and panic are the most prominent responses of individuals to illness in general, and these are exacerbated in catastrophic conditions [42,43]. In our opinion, in the era of COVID-19, the mental and physiologic recovery of the LT recipients may be very difficult. Furthermore, the reasons for this should be examined.

Depression in LT recipients ranges between 6.9% and 24.7% [44,45,46]. Risk factors of depression have been reported to be low monthly income, occurrence of biliary complications, patients having hepatocellular carcinoma as the indication of LT, and obligation to adhere to regular monthly out-patient clinic visits [34]. Akbulut et al. [47] have analyzed the factors affecting the depression, stress, and anxiety in patients with advanced HCC that did not receive LT and have shown that female gender, age, presence of chronic disease, low educational level, presence of smoking habit, and place of residence had significant impact in these patients. In the present study, analyses of the DASS-21 scale and its subdimensions showed a statistically significant difference between the scores obtained from the scales for the HCC and non-HCC groups, and it was determined that the total and subscale scores of the DASS-21 scale were significantly lower in the HCC group than in the non-HCC group. Furthermore, CAS-SF scores were also lower in the HCC group when compared to the non-HCC group. This is a very important finding because it suggests that anxiety, stress, and depression are more frequently observed in LT recipients who did not have HCC. Our experiences with the thousands of patients we have transplanted have shown that patients who are transplanted for HCC are concerned about the recurrence of their disease, which also has been shown in one of our previous studies. For this reason, they are more compliant to their postoperative surveillance program. Moreover, this results in an increased compliance rate concerning the isolation and protection methods that are recommended during the pandemic period. The results of the present study supports these arguments as well. Our literature search showed that there are no data regarding this subject. A study by Heo et al. [48] compared liver transplant recipients according to the etiology of liver failure, along with the incidence and severity of depression, in the pre- and posttransplant setting. Similar to our results, they found that patients with HCC were more resistant to the development and exacerbation of mental disorders in the posttransplant period. They also report that patients with substance abuse were more prone to developing mental disorders such as anxiety, somatization, and recurrence of substance abuse [48]. In our opinion, this emphasizes the impact of HCC on anxiety, stress, and mood disorders in patients. The presence of HCC is a factor causing depression and anxiety-related disorders, and our results show that these patients are not further affected by the stress factor created by the COVID-19 pandemic. However, this is a novel finding and should be confirmed by further studies on the subject.

A statistically significant difference was found for the scores of the DASS-21 scale and its subdimensions in terms of gender, and the DASS-21 total and subdimension scores of women were higher than those of men. This result shows that women react with more fear than do men. The CAS-SF scores were also considerably higher in female participants. This is a common finding in studies regarding depression. A population-based study on adolescents showed that the incidence of major depression in female adolescents and that they had worse course of major depression [49]. A meta-analysis in adults found that symptoms suggesting depression. such as rumination and reflection, were more prominent in females [50]. A study by Slavich et al. [51] showed that fluctuations in ovarian hormones may be one of the factors affecting the response of females to stress and result in a tendency to major depression. Similar results have been emphasized in other studies [52,53,54,55,56,57,58,59,60,61]. The effect of liver transplantation on the regulation of sex steroids and thus its effects on the major depressive symptoms need to be examined in further studies.

Our analyses showed that variables including presence of chronic disease, exposure to COVID-19 disease, vaccination for COVID-19, type of vaccine, hesitation to vaccinate for COVID-19, and belief in the efficacy of the COVID-19 vaccine showed significant differences in DASS-21 stress, DASS-21 depression, DASS-21 anxiety, and total DASS-21 scores. Briefly, it was observed in the current study that total and subdimension scores of DASS-21 were higher in those with chronic diseases, those who had COVID-19, those who did not have the COVID-19 vaccine, those who were hesitant about the vaccine, and those who stated that they had no idea about the protection of the vaccine and/or thought that it was not protective. In addition, the effect sizes ranged from small to large. However, the effect sizes obtained for the hesitation to vaccinate for COVID-19 were observed to be quite large. There was a clinically significant increase in the scores of the DASS-21 scale in those who were hesitant about the vaccine. Furthermore, according to the results obtained in the current study, CAS-SF scores were found to be higher in patients with chronic disease, women, postvaccine COVID-19 patients, those who had hesitations about the vaccine, and those who stated that they had no idea about the protection of the vaccine and/or thought that it was not protective. This is typical for the vaccine hesitancy in the current era and shows the lack of public information regarding the disease and the mechanism of action of the vaccines and their safety [62]. Our results also emphasize the importance of public education to promote the acceptance of vaccination.

It is known that psychological diseases have an effect on the health of the individuals. Anxiety and depression may cause chronic health problems or exacerbate the already existing conditions [63]. Depression, hopelessness, stress, and anxiety are risk factors for mental diseases in these individuals [64]. In addition, anxiety- and depression-related symptoms may impair the ability of LT recipients to adhere to postoperative adherence to immunosuppressive therapy and surveillance programs. This may have an impact on the survival of the patients [65]. In addition, the conditions associated with anxiety and depression in LT patients may be associated with conditions such as experiencing more negative effects of immunosuppressive drugs [66].

There are various limitations for the present study. Phone interview of the patients to fill the questionnaire forms may hinder the responses given by the individuals. Our experience shows that a face-to-face interview is the most ideal research method. However, it was not possible to use the face-to-face interview method in the COVID-19 pandemic. Moreover, the majority of our patients resided in other cities, and transportation to our institute was a major problem. The actual incidence and severity of depression may be higher than what we have found. Furthermore, the education level in Turkey is moderate, and the ability of individuals to understand these questionnaires may be limited as well. However, we tried our best to reduce the bias and data collection errors. To overcome this, expressions that the participant could understand were used during the phone interview. The results of our study are consistent with the current literature, and we have also brought some new insights regarding depression and anxiety disorders in liver transplant recipients.

In conclusion, we have shown increased COVID-19-related anxiety and depression in transplant recipients, and it can be concluded that clinical improvement of these conditions is very important in increasing their health. In addition, the results of the regression model revealed that chronic disease, gender, age, exposure to COVID-19, hesitation about the COVID-19 vaccine, and belief in the protection of the COVID-19 vaccine are effective in anxiety and depression. In addition, education of the public through conventional and social media has paramount importance in reducing anxiety-, stress-, and mood-related disorders in the public. This places considerable responsibility on the governments of countries around the world. Suitable educational adds and programs according to the sociodemographic and intellectual characteristics of the population should be produced to increase public awareness.

## Figures and Tables

**Table 1:** Comparison of the groups in terms of sociodemographic features.

Variables	HCC (n = 252)		Non-HCC (n = 252)		ES	*p*
	n	%	n	%		
Gender					0.255	<0.001 *
⠀⠀Female	39	28.9	96	71.1		
⠀⠀Male	213	57.7	156	42.3		
Marital Status					0.209	0.009 **
⠀⠀Married	237	51.9	220	48.1		
⠀⠀Single	15	31.9	32	68.1		
Educational Level					0.154	0.018 *
⠀⠀Illiterate ^a^	29	39.2	45	60.8		
⠀⠀Primary school ^a,b^	106	48.6	112	51.4		
⠀⠀Secondary school ^b^	55	65.5	29	34.5		
⠀⠀High school ^a,b^	43	47.3	48	52.7		
⠀⠀University ^a,b^	19	51.4	18	48.6		
Monthly Income					0.236	<0.001 *
⠀⠀Low ^a^	33	29.5	79	70.5		
⠀⠀Moderate ^b^	195	57.9	142	42.1		
⠀⠀Good ^b^	24	43.6	31	56.4		
Place of Residence					0.198	<0.001 *
⠀⠀City center ^a^	175	58.1	126	41.9		
⠀⠀District ^b^	54	38.0	88	62.0		
⠀⠀Village ^b^	23	37.7	38	62.3		
Smoking					0.005	0.905 *
⠀⠀Yes	43	50.6	42	49.4		
⠀⠀No	209	49.9	210	50.1		
Chronic disease					0.118	0.008 *
⠀⠀Yes	83	42.6	112	57.4		
⠀⠀No	169	54.7	140	45.3		

*: Pearson chi-square; **: Chi-square test with Yates correction. ^a,b^: statistically significant difference exists in group categories that do not contain the same letter.

**Table 2:** Comparison of the groups in terms of COVID-19-related features.

Variables	HCC (n = 252)		Non-HCC (n = 252)		ES	*p*
	n	%	n	%		
Exposure to COVID-19					0.028	0.530 *
⠀⠀Yes	63	52.5	57	47.5		
⠀⠀No	189	49.2	195	50.8		
Need for Hospitalization Due to COVID-19					0.098	0.620 **
⠀⠀Yes	18	60.0	12	40.0		
⠀⠀No	42	48.0	44	51.2		
Vaccination for COVID-19					0.099	0.026 *
⠀⠀Yes	217	52.3	198	47.7		
⠀⠀No	35	39.3	54	60.7		
COVID-19 Vaccine Doses					0.187	0.001 *
⠀⠀1 dose ^a^	13	27.7	34	72.3		
⠀⠀2 doses ^a,b^	103	52.3	94	47.7		
⠀⠀3 doses ^a,b^	101	59.1	70	40.9		
Type of COVID-19 Vaccine					0.139	0.019 *
⠀⠀Sinovac™ ^a,b^	89	48.6	94	51.4		
⠀⠀Biontech™ ^a,b^	64	48.1	69	51.9		
⠀⠀Both ^a^	64	64.6	35	35.4		
Postvaccination COVID-19 Exposure					0.063	0.259 **
⠀⠀Yes	16	42.1	22	57.9		
⠀⠀No	203	53.3	179	46.7		
Hesitancy to Vaccinate for COVID-19					0.009	0.846 *
⠀⠀Yes	74	49.3	76	50.7		
⠀⠀No	178	50.3	176	49.7		
Belief in the Efficacy of the COVID-19 Vaccine					0.128	0.016 *
⠀⠀Yes ^a^	192	53.3	168	46.7		
⠀⠀No ^b^	10	29.4	24	70.6		
⠀⠀No idea ^a,b^	50	45.5	60	54.5		
Approval of the Mandatory Vaccination for COVID-19					0.259	<0.001 *
⠀⠀Yes ^a^	139	47.4	154	52.6		
⠀⠀No ^b^	11	20.4	43	79.6		
⠀⠀No idea ^c^	102	65.0	55	35.0		

* Pearson chi-square; ** Chi-square test with Yates correction. ^a,b,c^: statistically significant difference exists in group categories that do not contain the same letter.

**Table 3:** Comparison of the independent variables according to the subdimension and total scores of the DASS-21 scale.

Variables [Median (IQR)]	DASS-21 Stress				DASS-21 Anxiety				DASS-21 Depression				DASS-21 Total			
	Med.	IQR	ES	*p*	Med.	IQR	ES	*p*	Med.	IQR	ES	*p*	Med.	IQR	ES	*p*
Group			0.281	0.002 *			0.535	<0.001 *			0.309	<0.001 *			0.398	<0.001 *
⠀⠀HCC	1.0	3.50			0.0	1.00			0.0	2.00			3.0	6.00		
⠀⠀Non-HCC	2.0	3.00			1.0	2.00			1.0	3.00			4.0	7.00		
Gender			0.188	0.032 *			0.424	<0.001 *			0.268	0.001 *			0.284	0.001 *
⠀⠀Female	2.0	3.00			1.0	3.00			1.0	4.00			5.0	9.00		
⠀⠀Male	2.0	4.00			0.0	1.00			0.0	2.00			3.0	5.00		
Chronic Disease			0.356	<0.001 *			0.307	<0.001 *			0.224	0.007 *			0.418	<0.001 *
⠀⠀Yes	3.0	3.00			1.0	2.00			1.0	3.00			5.0	7.00		
⠀⠀No	1.0	4.00			0.0	1.00			0.0	2.00			3.0	6.00		
Exposure to COVID-19			0.38	<0.001 *			0.196	0.017 *			0.311	<0.001 *			0.394	<0.001 *
⠀⠀Yes	3.0	4.50			1.0	3.00			1.0	4.00			6.0	9.50		
⠀⠀No	2.0	3.00			0.0	2.00			0.0	2.00			3.0	5.00		
Vaccination for COVID-19			0.235	0.008 *			0.162	0.048 *			0.221	0.008 *			0.266	0.003 *
⠀⠀Yes	2.0	4.00			0.0	2.00			0.0	2.00			4.0	6.00		
⠀⠀No	3.0	3.00			1.0	3.00			1.0	4.00			4.0	9.00		
Type of Vaccine			0.306	0.003 **			0.236				0.399	<0.001 **			0.387	<0.001 **
⠀⠀Sinovac™	2.0 ^a^	4.00			0.0 ^a^	2.00		0.021 **	1.0 ^a^	3.00			4.0 ^a^	7.00		
⠀⠀Biontech™	1.0 ^a^	4.00			0.0 ^ab^	2.00			0.0 ^a^	2.00			4.0 ^a^	6.00		
⠀⠀Both *	1.0 ^b^	2.00			0.0 ^b^	1.00			0.0 ^b^	1.00			2.0 ^b^	5.00		
Hesitancy to Vaccinate for COVID-19			1.589	<0.001 *			1.634	<0.001 *			1.492	0.004 *			1.605	<0.001 *
⠀⠀Yes	3.0	3.00			1.0	3.00			1.0	3.00			5.0	8.00		
⠀⠀No	1.0	4.00			0.0	1.00			0.0	2.00			3.0	6.00		
Belief in Efficacy of Vaccination for COVID-19			0.171	<0.001 **			0.279	<0.001 **			0.273	<0.001 **			0.238	<0.001 **
⠀⠀Yes	1.0 ^a^	4.00			0.0 ^a^	1.00			0.0 ^a^	2.00			3.0 ^a^	7.00		
⠀⠀No	3.0 ^b^	3.00			1.5 ^b^	4.00			3.0 ^b^	5.00			7.0 ^b^	11.0		
⠀⠀No idea	2.0 ^b^	3.00			1.0 ^b^	3.00			1.0 ^c^	3.00			4.5 ^c^	7.00		

* Mann–Whitney U test; ** Kruskal–Wallis test. ^a,b,c^: statistically significant difference exists in group categories that do not contain the same letter.

**Table 4:** Comparison of some independent variables according to CAS-SF scores.

Variables [Median (IQR)] [Mean and SD]	CAS-SF				ES	*p*
	Median	IQR	Mean	SD		
Group						
⠀⠀HCC	0.0	0.0	0.64	1.64	0.22	0.002 *
⠀⠀Non-HCC	0.0	1.0	0.61	1.00		
Gender					0.246	0.001 *
⠀⠀Female	0.0	1.0	0.86	1.52		
⠀⠀Male	0.0	1.0	0.55	1.30		
Chronic Disease					0.214	0.003 *
⠀⠀Yes	0.0	1.0	0.77	1.38		
⠀⠀No	0.0	1.0	0.54	1.35		
Exposure to COVID-19					0.071	0.325 *
⠀⠀Yes	0.0	1.0	0.90	1.96		
⠀⠀No	0.0	1.0	0.55	1.11		
Need for Hospitalization Due to COVID-19					0.146	0.351 *
⠀⠀Yes	0.0	1.0	0.97	2.46		
⠀⠀No	0.0	1.0	0.87	1.77		
Vaccination for COVID-19					0.054	0.458 *
⠀⠀Yes	0.0	1.0	0.65	1.39		
⠀⠀No	0.0	1.0	0.56	1.25		
COVID-19 Vaccination Dose					0.119	0.760 **
⠀⠀1 dose	0.0	1.0	0.72	1.62		
⠀⠀2 doses	0.0	1.0	0.64	1.36		
⠀⠀3 doses	0.0	1.0	0.63	1.38		
Type of vaccine					0.093	0.573 **
⠀⠀Sinovac™	0.0	1.0	0.70	1.52		
⠀⠀Biontech™	0.0	1.0	0.58	1.37		
⠀⠀Both	0.0	1.0	0.62	1.17		
Postvaccination COVID-19 Exposure					0.194	0.015 *
⠀⠀Yes	0.0	2.0	1.16	2.07		
⠀⠀No	0.0	1.0	0.59	1.30		
Hesitancy to Vaccinate for COVID-19					0.239	0.001 *
⠀⠀Yes	0.0	1.0	0.87	1.62		
⠀⠀No	0.0	1.0	0.53	1.24		
Belief in the Efficacy of COVID-19 Vaccines					0.234	0.013 **
⠀⠀Yes	0.0	1.0	0.56	1.30		
⠀⠀No	0.0	1.0	0.94	1.67		
⠀⠀No idea	0.0	1.0	0.77	1.46		
Approval of Mandatory Vaccination for COVID-19					0.11	0.173 **
⠀⠀Yes	0.0	1.0	0.50	1.06		
⠀⠀No	0.0	1.0	0.74	1.25		
⠀⠀No idea	0.0	1.0	0.83	1.82		

*: Mann–Whitney U test; **: Kruskal–Wallis rest.

**Table 5:** The results of the reliability analyses and correlations of the scales.

		DASS-21 Stress	DASS-21 Anxiety	DASS-21 Depression	CAS-SF	DASS-21 Total
						α
DASS-21 Stress (7 items)	r	1.000				0.823
	*p*	-----				
	α	0.766				
DASS-21 Anxiety (7 items)	r	0.499	1.000			
	*p*	<0.001	-----			
	α	-----	0.776			
DASS-21 Depression (7 items)	r	0.504	0.513	1.000		
	*p*	<0.001	<0.001	-----		
	α	-----	-----	0.859		
CAS-SF (5 items)	r	0.148	0.292	0.325	1.000	
	*p*	0.001	<0.001	<0.001	-----	
	α	-----	-----	-----	0.783	

**Table 6:** Results of the negative binomial regression model for total scores of the DASS-21 scale.

Variables	Categories	IRR	95% CI	*p*
Group	[1: HCC; 2: Non-HCC]	1.26	1.01–1.52	0.031
Age	-	1.01	1.01–1.03	0.008
Gender	[1: Female; 2: Male]	1.29	1.04–1.69	0.036
Educational Level		1.00	0.91–1.11	0.969
Monthly Income	[1: Poor; 2: Moderate; 3: Good]	1.19	1.00–1.47	0.069
Smoking	[1: Yes; 2: No]	1.19	0.95–1.64	0.186
Chronic Disease	[1: Yes; 2: No]	1.65	1.39–2.08	<0.001
COVID-19 Exposure	[1: Yes; 2: No]	1.63	1.35–2.13	<0.001
Vaccination for COVID-19	[1: Yes; 2: No]	1.50	1.11–1.87	0.002

IRR: incidence rate ratio; CI: confidence interval; HCC: hepatocellular carcinoma.

**Table 7:** Results of the negative binomial regression model for CAS-SF.

Variables	Categories	IRR	95% CI	*p*
Group	[1: HCC; 2: Non-HCC]	1.33	0.95–2.27	0.160
Age	-	1.00	0.98–1.02	0.728
Gender	[1: Female; 2: Male]	1.75	1.22–3.22	0.014
Educational Level		1.09	0.89–1.29	0.348
Monthly Income	[1: Poor; 2: Moderate; 3: Good]	1.36	1.00–2.12	0.090
Smoking	[1: Yes; 2: No]	1.15	0.77–2.38	0.579
Chronic Disease	[1: Yes; 2: No]	1.38	1.00–2.27	0.107
COVID-19 Exposure	[1: Yes; 2: No]	1.51	1.07–2.63	0.048
Vaccination for COVID-19	[1: Yes; 2: No]	1.09	0.72–1.39	0.731

IRR: incidence rate ratio; CI: confidence interval; HCC: hepatocellular carcinoma.

**Table 8:** Variable importance values obtained from the MLP model for DASS-21 total.

Variables	Importance
Exposure to COVID-19	0.269
Group	0.187
Belief in the Efficacy of the COVID-19 Vaccine	0.179
Hesitancy to Vaccinate for COVID-19	0.141
Chronic Disease	0.127
Vaccination for COVID-19	0.071
Gender	0.026

**Table 9:** Variable importance values obtained from the MLP model for CAS-SF.

Variables	Importance
Chronic Disease	0.309
Group	0.219
Hesitancy to Vaccinate for COVID-19	0.185
Belief in the Efficacy of the COVID-19 Vaccine	0.147
Gender	0.098
Vaccination for COVID-19	0.040

## Data Availability

The datasets analyzed during the current study are available from the corresponding author on reasonable request.

## Abbreviations

HCC—hepatocellular carcinoma; HBV—hepatitis B virus; HCV—hepatitis C virus; NAFLD—nonalcoholic fatty liver disease; CLD—chronic liver disease; RFA—radio-frequency ablation; MWA—microwave ablation; HIFU—high-intensity focused ultrasound; IRE—irreversible electroporation; TACE—transcatheter arterial chemoembolization; TARE—transcatheter arterial radioembolization; LT—liver transplantation; STROBE—Strengthening the Reporting of Observational Studies in Epidemiology; DASS—Depression, Stress and Anxiety Scale; CAS—Coronavirus Anxiety Scale; SPSS—Statistical Package for the Social Sciences; IRR—incidence rate ratio.
